# Supplementary material for: ADAR1-mediated regulation of melanoma invasion
Source: Nat Commun. 2018 May 31;9:2154. doi: 10.1038/s41467-018-04600-2 (PMC5981216; doi:10.1038/s41467-018-04600-2)
Supplement: Supplementary file 1 — Supplementary Information [file 41467_2018_4600_MOESM1_ESM.pdf]

Supplementary Figure 1: Central position for ITGB3 among the invasion and migration functionally related genes

A list of 100 genes which their expression was altered due to ADAR1 silencing and functionally involved in cellular invasion and/or migration were examined using an online protein-protein interactions analysis tool - "String". 85 out of 100 (85%) genes were found to be functionally linked, while ITGB3 is located at the center of the protein network.

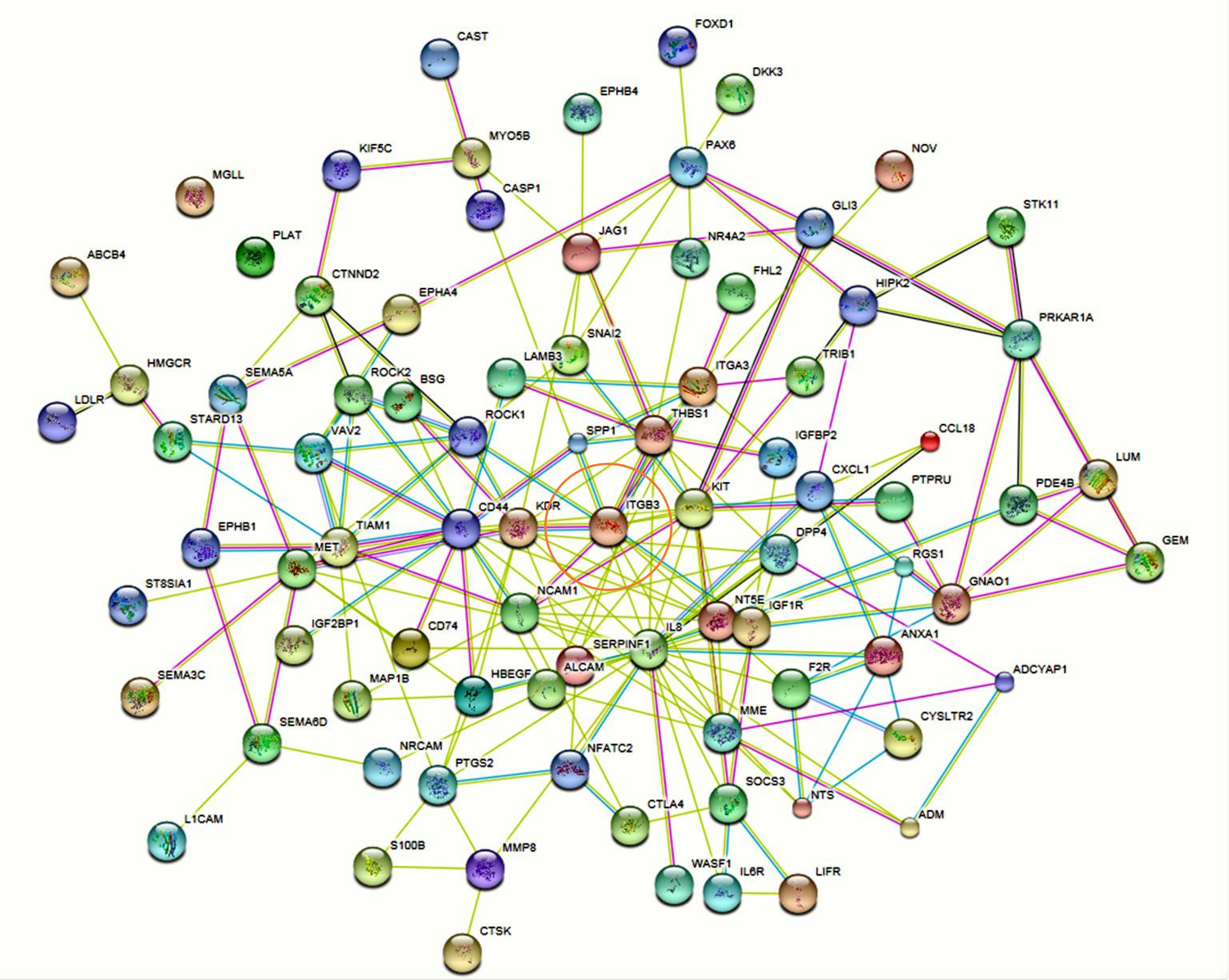

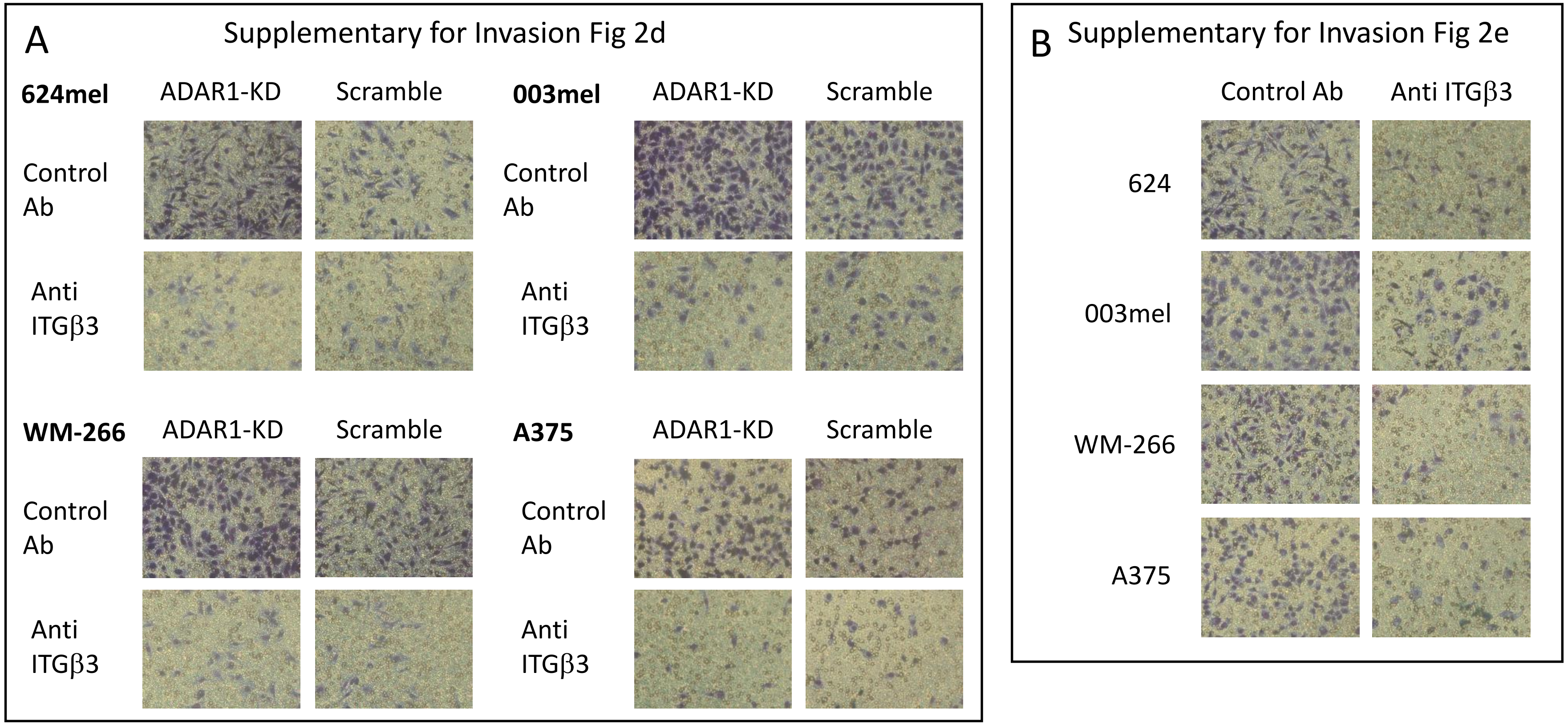

Supplementary Figure 2(A-B): Microphotographs of invading cells fixed on representative transwell membranes. All invasion experiments were performed using XTT quantification and by membrane fixation followed by Giemza staining. Representative membranes (out of triplicates photographed from each experiment) are shown here for all experiments. The data supplements the corresponding figures as indicated.

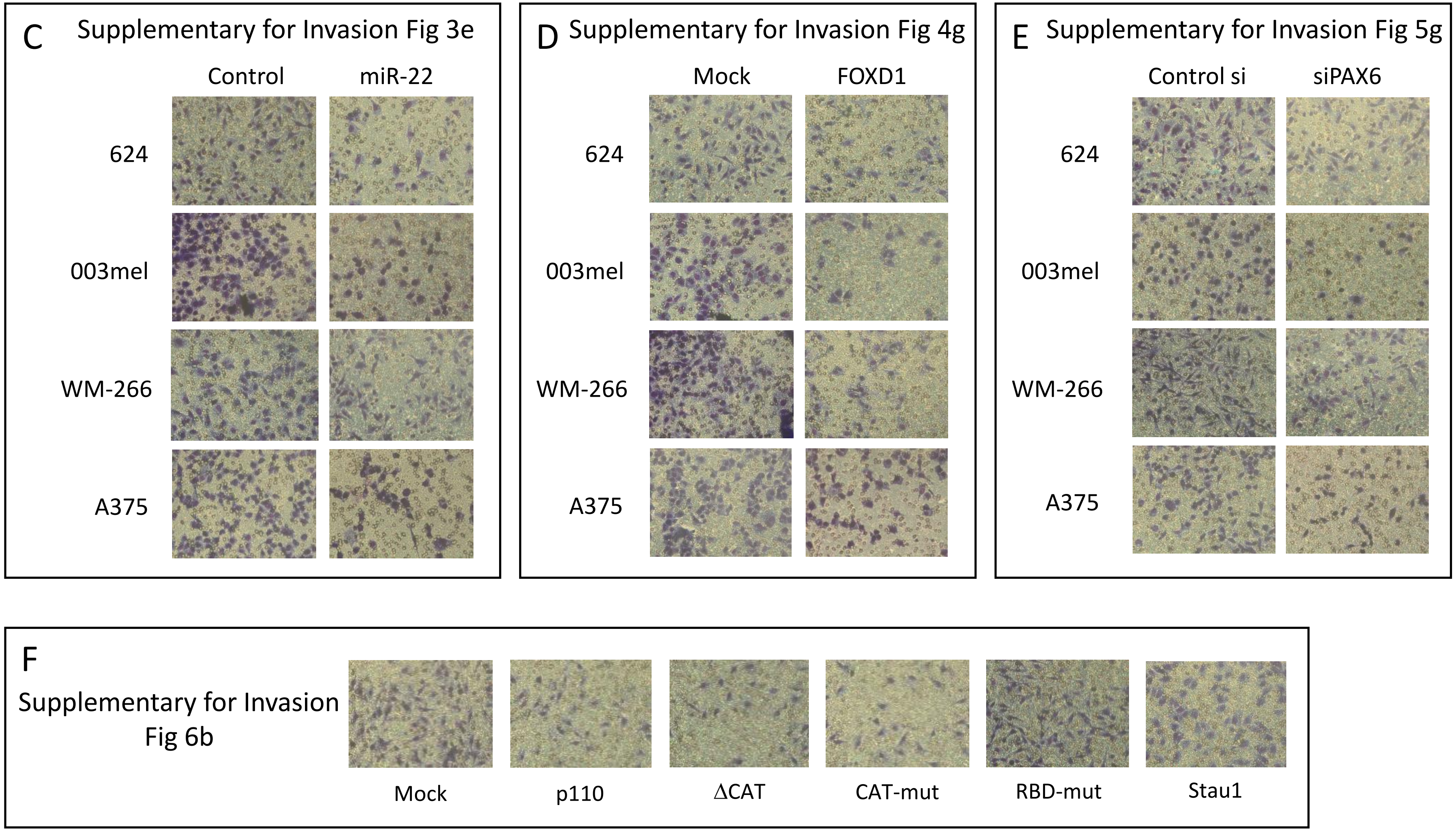

Supplementary Figure 2(C-F): Microphotographs of invading cells fixed on representative transwell membranes. All invasion experiments were performed using XTT quantification and by membrane fixation followed by Giemza staining. Representative membranes (out of triplicates photographed from each experiment) are shown here for all experiments. The data supplements the corresponding figures as indicated.

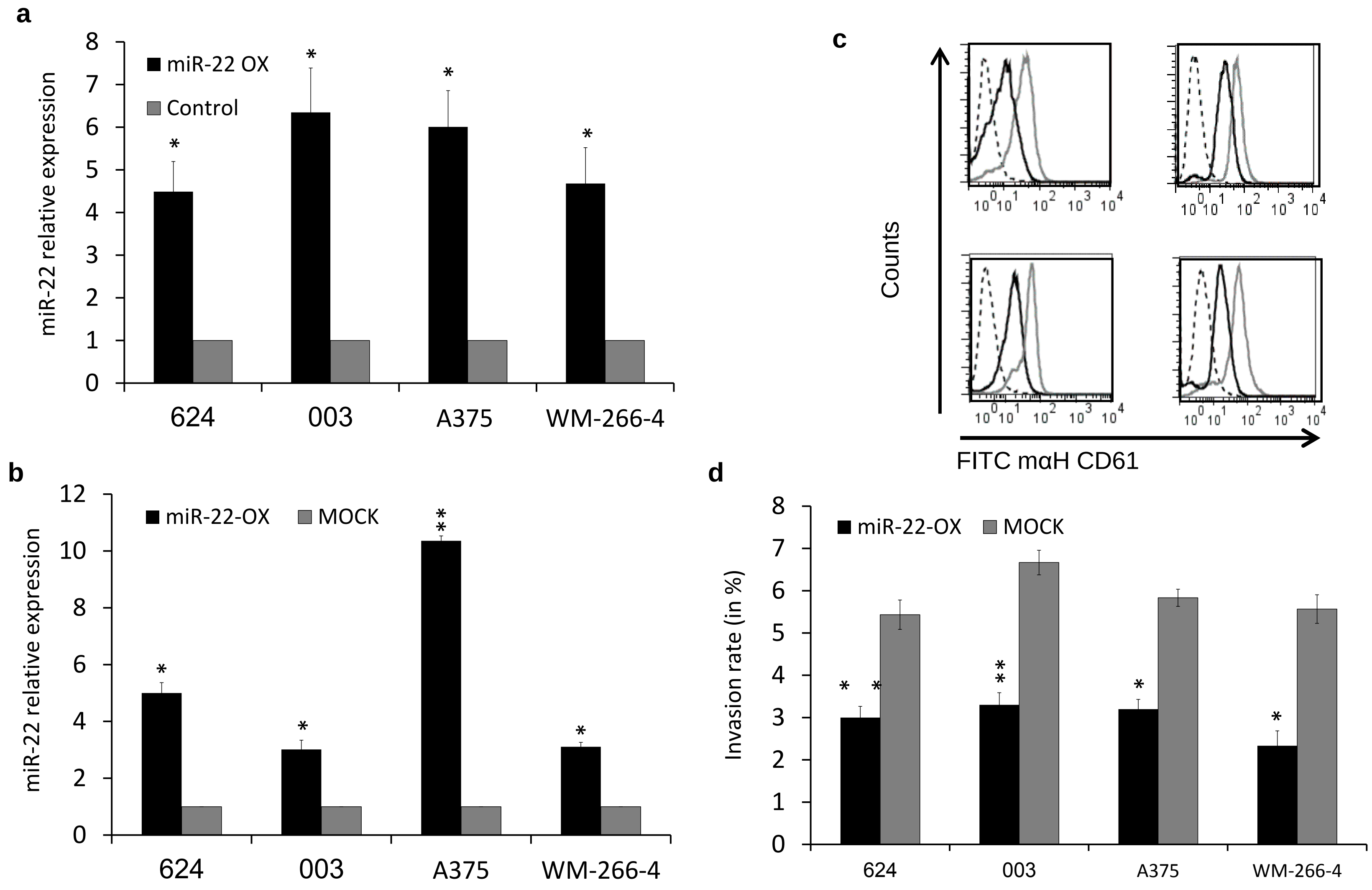

Supplementary Figure 3: Regulation of ITGB3 expression and function by miR-22.(a) Mature miR-22 expression in 4 melanoma lines following transient transfection of miR-22 mimic (mir-22 OX) or control oligo (Control) was examined by qRT-PCR; (b) Pri-miR-22 was cloned into pQCXIP plasmid (miR-22-OX) and negative control (empty vector, Mock) were stably transduced into 4 melanoma lines: 624, 003, A375 and WM-266-4. Expression of mature miR-22 in the miR-22-OX as compared to control was confirmed using qRT-PCR; (c) ITGB3 expression was examined using extracellular FACS staining with an ITGB3 FITC conjugated antibody. The effect of miR-22-OX (gray line) is compared to Mock (black line). Dashed line depicts Isotype control; (d) Invasion was tested using Boyden chamber assay and monitored by standardized XTT assay, for 24hrs. The effect of miR-22-OX (black bars) is compared to Mock (gray bars). Results for represent the mean ±SE of 3 biologically independent experiments, each performed in triplicates. Asterisks represent P values: *P<0.05, **P<0.01, ***P<0.001 (2-tailed t-test).

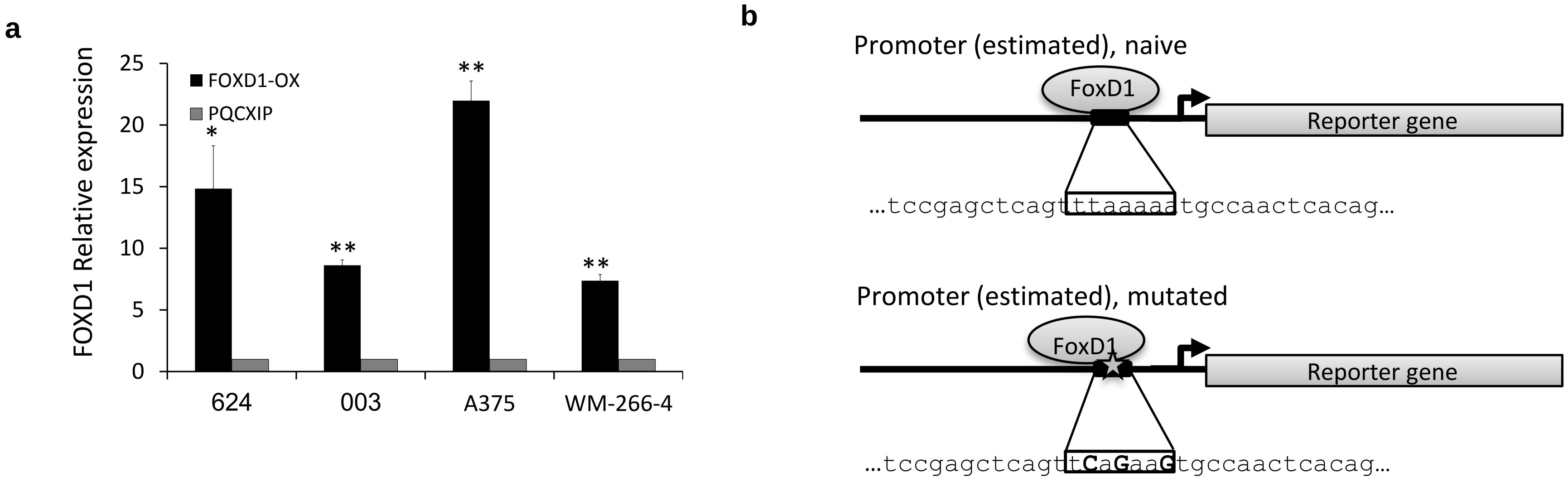

Supplementary Figure 4: FoxD1 and miR-22 promoter. (a)The expression of FoxD1 in four melanoma cell lines that were stably transduced with FoxD1 over-expression plasmid (FoxD1-OX) or Control (Mock) plasmid was examined by qRT-PCR. Results for represent the mean ±SE of 3 biologically independent experiments, each performed in triplicates. Asterisks represent P values: *P<0.05, **P<0.01, (2-tailed t-test); (b) Schematic representation of the miR-22HG promoter region fused to the luciferase reporter gene and its predicted FoxD1 binding site. The naïve binding site (upper panel) and mutated binding site (lower panel) are detailed.

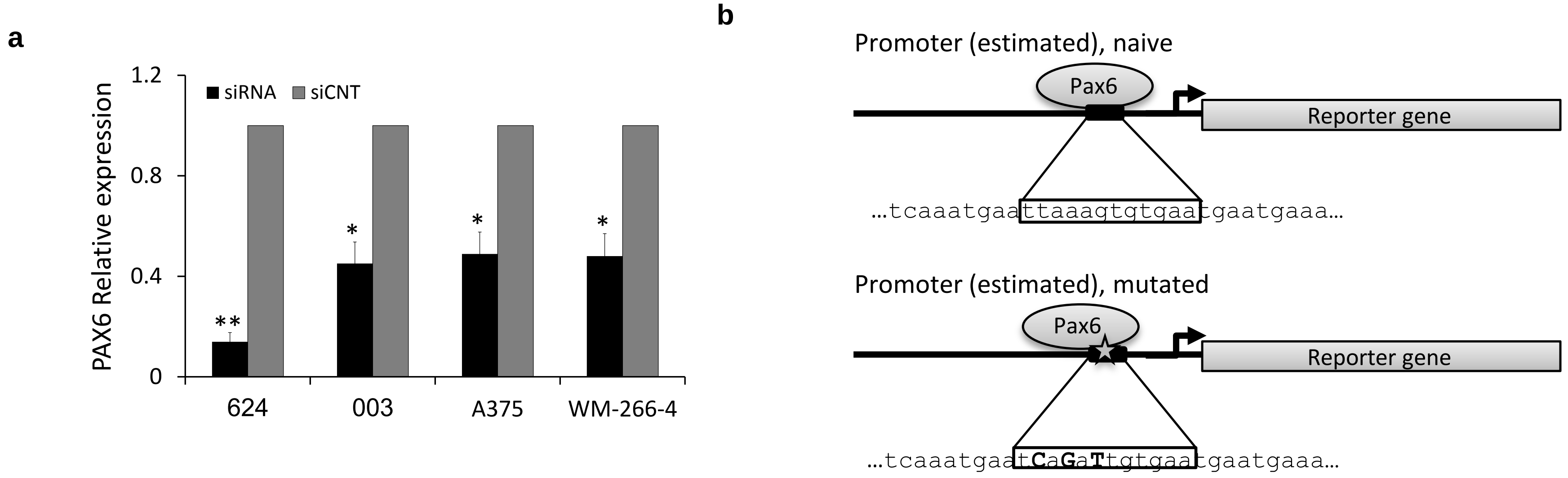

Supplementary Figure 5: Pax6 and ITGB3 promoter. (a) The expression of PAX6 in four melanoma cell lines that were transfected with PAX6 siRNA (siPAX6) or Control siRNA (siCNT) was examined by qRT-PCR. Results for represent the mean ±SE of 3 biologically independent experiments, each performed in triplicates. Asterisks represent P values: *P<0.05, **P<0.01, (2-tailed t-test); (b) Schematic representation of the ITGB3 promoter region fused to the luciferase reporter gene and its predicted PAX6 binding site. The naïve binding site (upper panel) and mutated binding site (lower panel) are detailed.

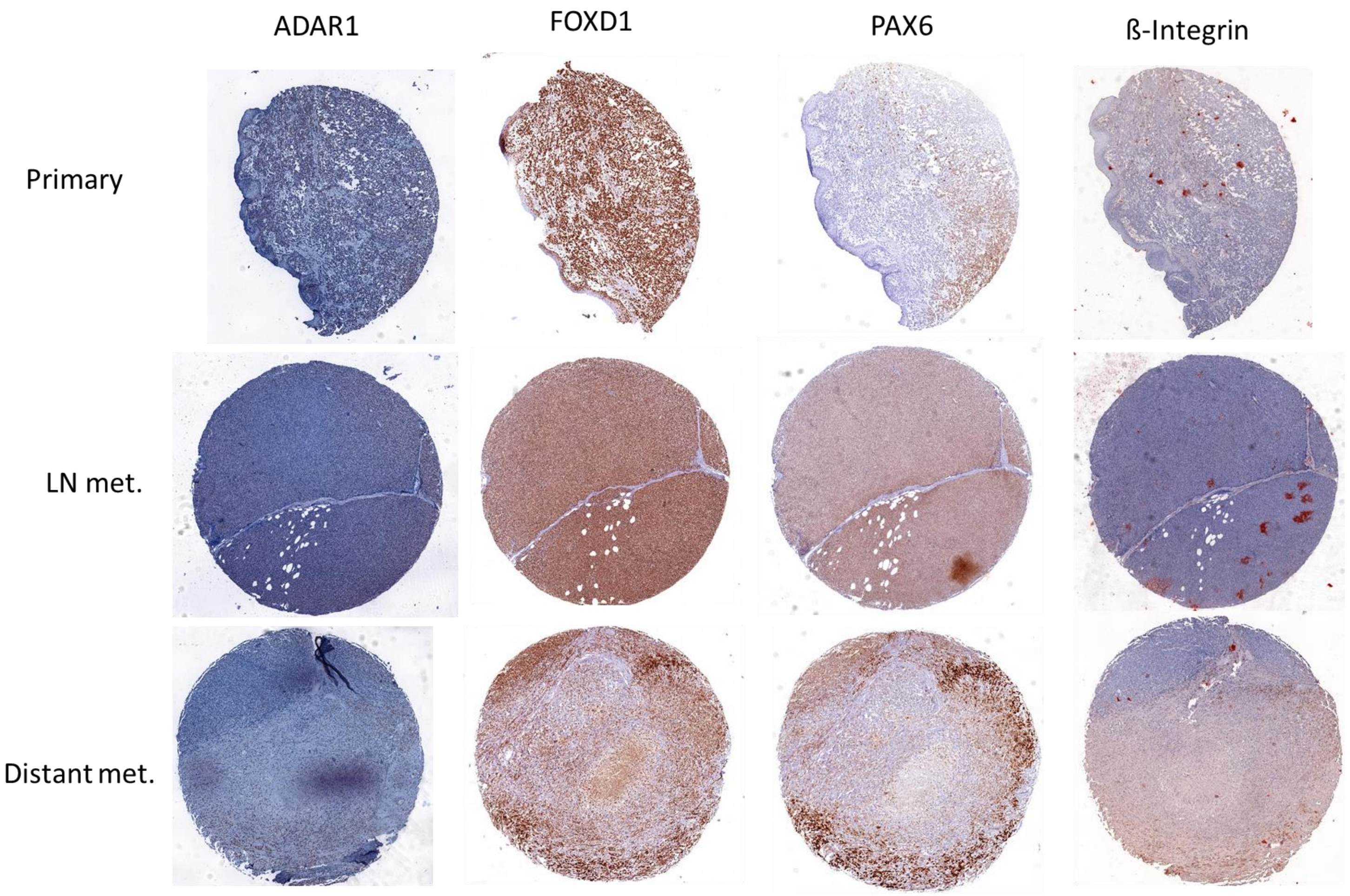

Supplementary Figure 6 –ADAR1-FOXD1-PAX6-ITGb3 pathway during melanoma progression in a rrepresentative patient.

Patient-paired progression tissue microarray was generated from 12 patients from which tissue specimens at all disease stages were available (primary, lymph node metastasis and distant metastasis). This figure shows a x4 magnification of entire cores, which enables appreciation of the general staining results. All cores are from a single representative patient, which enables true evaluation of disease progression.

a

GGCUGAGCCGC**A**GUAGUUCUUC**A**GUGGCAAGCUUUAUGUCCUGACCC**A**GCU**AAA**GCUGCC**A**GUUGA**A**GA**A**C
UGUUGCCCUCUGCC

b

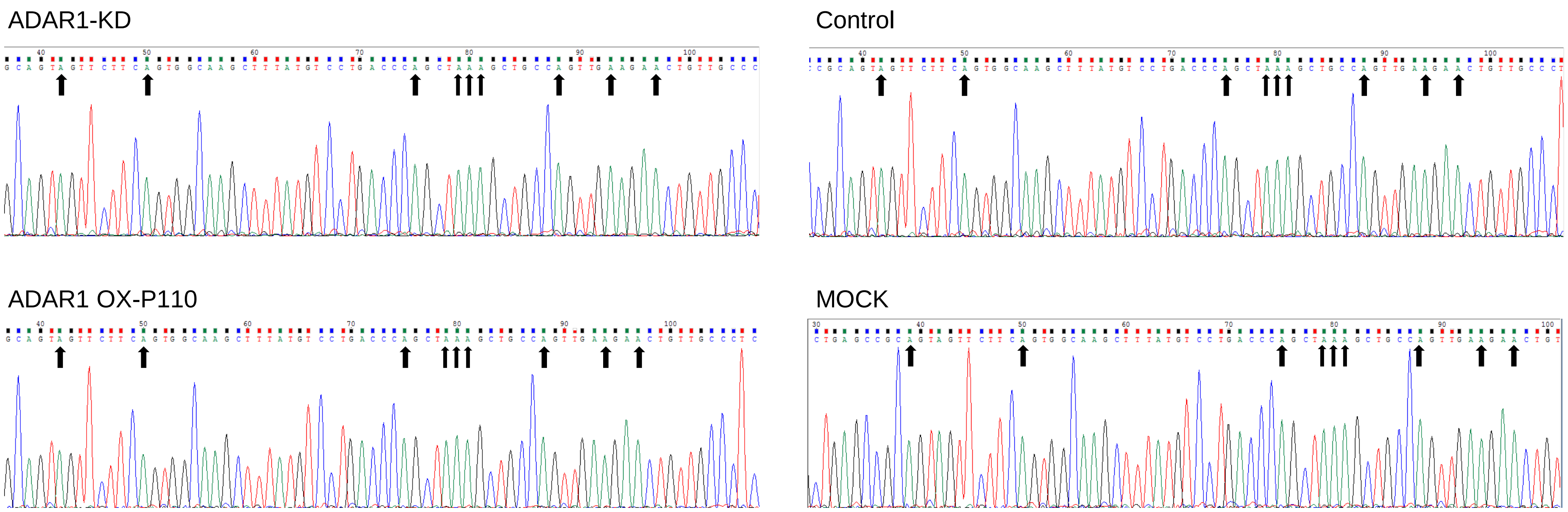

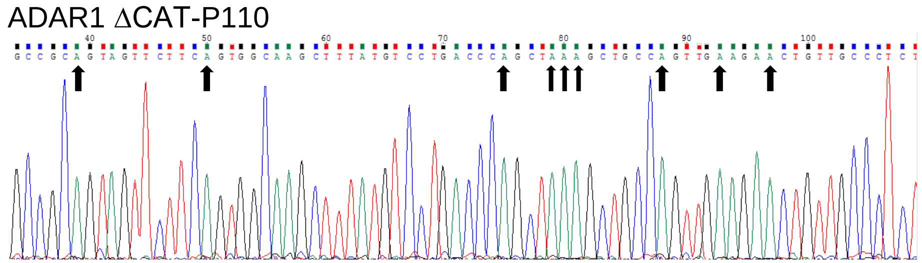

Supplementary Figure 7: miR-22 editing. (a) The predicted locations of A to I editing sites is marked with black arrows. The mature miR-22 sequence is underlined. (b) Representative Sequencing results of mir-22 mRNA of ADAR1 "Domains" cell system: OX-P110, CAT-MUT-P110, ΔCAT-P110 and Mock. Direct sequencing of pri-miR-22 PCR products with the appropriate primers was performed on RNA/cDNA originated from 3 biologically independent systems. The corresponding A's to the predicted editing sites are marked with black arrows

Supplementary Table 1

**Patient number**	**Age**	**Gender**	**Location of primary tumor**	**Location of lymph node metastases**	**Location of distant metastases**
**1**	66	F	Vulva	Inguinal	Small bowel
**2**	70	F	Upper back	Axilla	Sub-cutaneous
**3**	83	F	Leg	Thigh	Sub-cutaneous
**4**	34	M	Leg	Inguinal	Sub-cutaneous
**5**	59	M	Conjunctiva	Neck	Retroperitoneum
**6**	60	M	Flank	Axilla	Supraclavicular LN
**7**	71	M	Shoulder	Axilla	Muscle
**8**	62	F	Flank	Axilla	Sub-cutaneous
**9**	51	F	Buttocks	Inguinal	Sub-cutaneous
**10**	43	F	Vagina	Inguinal	Sub-cutaneous
**11**	85	M	Scalp	Neck	Salivary gland
**12**	50	F	Vulva	Inguinal	Lung
