# Supplementary material for: ADAR1-mediated regulation of melanoma invasion
Source: Nat Commun. 2018 May 31;9:2154. doi: 10.1038/s41467-018-04600-2 (PMC5981216; doi:10.1038/s41467-018-04600-2)
Supplement: Supplementary file 3 — Description of Additional Supplementary Files [file 41467_2018_4600_MOESM3_ESM.pdf]

Description of Additional Supplementary Files

File Name: Supplementary Data 1
Description: Depicts the full list of differentially expressed genes following ADAR1 knockdown that are associated with invasion.

File Name: Supplementary Data 2
Description: Depicts the sequences of all of the primers used in this study.
