# Supplementary material for: ADAR1-mediated regulation of melanoma invasion
Source: Nat Commun. 2018 May 31;9:2154. doi: 10.1038/s41467-018-04600-2 (PMC5981216; doi:10.1038/s41467-018-04600-2)
Supplement: Supplementary file 4 — Supplementary Data 1 [file 41467_2018_4600_MOESM4_ESM.docx]

**Supplementary Data 1: Invasion and migration gene list**

| **Gene symbol** | **Gene name** | **Accession No.** | **Fold Change** |
| --- | --- | --- | --- |
| JAG1 | jagged 1 (Alagille syndrome) | [NM_000214](http://www.ncbi.nlm.nih.gov/entrez/query.fcgi?db=nucleotide&cmd=search&term=NM_000214) | 0.1 |
| THBS1 | thrombospondin 1 | [NM_003246](http://www.ncbi.nlm.nih.gov/entrez/query.fcgi?db=nucleotide&cmd=search&term=NM_003246) | 0.1 |
| ABCB4 | ATP-binding cassette, sub-family B (MDR | [NM_018849](http://www.ncbi.nlm.nih.gov/entrez/query.fcgi?db=nucleotide&cmd=search&term=NM_018849) | 0.2 |
| LIFR | leukemia inhibitory factor receptor alpha | [NM_002310](http://www.ncbi.nlm.nih.gov/entrez/query.fcgi?db=nucleotide&cmd=search&term=NM_002310) | 0.2 |
| SERPINA3 | serpin peptidase inhibitor, clade A (alpha-1 antiproteinase, antitrypsin), member 3 | [NM_001085](http://www.ncbi.nlm.nih.gov/entrez/query.fcgi?db=nucleotide&cmd=search&term=NM_001085) | 0.2 |
| SOCS3 | suppressor of cytokine signaling 3 | [NM_003955](http://www.ncbi.nlm.nih.gov/entrez/query.fcgi?db=nucleotide&cmd=search&term=NM_003955) | 0.2 |
| SPP1 | secreted phosphoprotein 1 (osteopontin, bone sialoprotein I, early T-lymphocyte activation 1) | [NM_001040060](http://www.ncbi.nlm.nih.gov/entrez/query.fcgi?db=nucleotide&cmd=search&term=NM_001040060) | 0.2 |
| CTNND2 | catenin (cadherin-associated protein), delta 2 (neural plakophilin-related arm-repeat protein) | [NM_001332](http://www.ncbi.nlm.nih.gov/entrez/query.fcgi?db=nucleotide&cmd=search&term=NM_001332) | 0.3 |
| HIPK2 | homeodomain interacting protein kinase 2 | [NM_022740](http://www.ncbi.nlm.nih.gov/entrez/query.fcgi?db=nucleotide&cmd=search&term=NM_022740) | 0.3 |
| HOXA7 | homeobox A7 | [NM_006896](http://www.ncbi.nlm.nih.gov/entrez/query.fcgi?db=nucleotide&cmd=search&term=NM_006896) | 0.3 |
| MET | met proto-oncogene (hepatocyte growth factor receptor) | [NM_000245](http://www.ncbi.nlm.nih.gov/entrez/query.fcgi?db=nucleotide&cmd=search&term=NM_000245) | 0.3 |
| SEMA5A | sema domain, seven thrombospondin repeats transmembrane domain ™ and short cytoplasmic domain, (rystalline) 5A | [NM_003966](http://www.ncbi.nlm.nih.gov/entrez/query.fcgi?db=nucleotide&cmd=search&term=NM_003966) | 0.3 |
| STARD13 | START domain containing 13 | [NM_178007](http://www.ncbi.nlm.nih.gov/entrez/query.fcgi?db=nucleotide&cmd=search&term=NM_178007) | 0.3 |
| ABLIM1 | actin binding LIM protein 1 | [NM_001003408](http://www.ncbi.nlm.nih.gov/entrez/query.fcgi?db=nucleotide&cmd=search&term=NM_001003408) | 0.4 |
| ADM | adrenomedullin | [NM_001124](http://www.ncbi.nlm.nih.gov/entrez/query.fcgi?db=nucleotide&cmd=search&term=NM_001124) | 0.4 |
| ALCAM | activated leukocyte cell adhesion molecule | [NM_001627](http://www.ncbi.nlm.nih.gov/entrez/query.fcgi?db=nucleotide&cmd=search&term=NM_001627) | 0.4 |
| GLI3 | GLI-Kruppel family member GLI3 (Greig cephalopolysyndactyly syndrome) | [NM_000168](http://www.ncbi.nlm.nih.gov/entrez/query.fcgi?db=nucleotide&cmd=search&term=NM_000168) | 0.4 |
| GNAO1 | guanine nucleotide binding protein (G protein), alpha activating activity polypeptide O | [NM_020988](http://www.ncbi.nlm.nih.gov/entrez/query.fcgi?db=nucleotide&cmd=search&term=NM_020988) | 0.4 |
| IL6R | interleukin 6 receptor | [NM_000565](http://www.ncbi.nlm.nih.gov/entrez/query.fcgi?db=nucleotide&cmd=search&term=NM_000565) | 0.4 |
| IL8 | interleukin 8 | [NM_000584](http://www.ncbi.nlm.nih.gov/entrez/query.fcgi?db=nucleotide&cmd=search&term=NM_000584) | 0.4 |
| ITGA3 | integrin, alpha 3 (antigen CD49C, alpha 3 subunit of VLA-3 receptor) | [NM_002204](http://www.ncbi.nlm.nih.gov/entrez/query.fcgi?db=nucleotide&cmd=search&term=NM_002204) | 0.4 |
| MME | membrane metallo-endopeptidase (neutral endopeptidase, enkephalinase) | [NM_007288](http://www.ncbi.nlm.nih.gov/entrez/query.fcgi?db=nucleotide&cmd=search&term=NM_007288) | 0.4 |
| NT5E | 5'-nucleotidase, ecto (CD73) | [NM_002526](http://www.ncbi.nlm.nih.gov/entrez/query.fcgi?db=nucleotide&cmd=search&term=NM_002526) | 0.4 |
| PAEP | progestagen-associated endometrial protein (placental protein 14, pregnancy-associated endometrial alpha-2-globulin, alpha uterine protein) | [NM_001018049](http://www.ncbi.nlm.nih.gov/entrez/query.fcgi?db=nucleotide&cmd=search&term=NM_001018049) | 0.4 |
| PTGS2 | prostaglandin-endoperoxide synthase 2 (prostaglandin G | [NM_000963](http://www.ncbi.nlm.nih.gov/entrez/query.fcgi?db=nucleotide&cmd=search&term=NM_000963) | 0.4 |
| PTPRU | protein tyrosine phosphatase, receptor type, U | [NM_133178](http://www.ncbi.nlm.nih.gov/entrez/query.fcgi?db=nucleotide&cmd=search&term=NM_133178) | 0.4 |
| RLN2 | relaxin 2 | [NM_005059](http://www.ncbi.nlm.nih.gov/entrez/query.fcgi?db=nucleotide&cmd=search&term=NM_005059) | 0.4 |
| SEMA6D | sema domain, transmembrane domain ™, and cytoplasmic domain, (rystalline) 6D | [NM_153618](http://www.ncbi.nlm.nih.gov/entrez/query.fcgi?db=nucleotide&cmd=search&term=NM_153618) | 0.4 |
| STK11 | serine | [NM_000455](http://www.ncbi.nlm.nih.gov/entrez/query.fcgi?db=nucleotide&cmd=search&term=NM_000455) | 0.4 |
| BSG | basigin (Ok blood group) | [NM_001728](http://www.ncbi.nlm.nih.gov/entrez/query.fcgi?db=nucleotide&cmd=search&term=NM_001728) | 0.5 |
| CXCL1 | chemokine (C-X-C motif) ligand 1 (melanoma growth stimulating activity, alpha) | [NM_001511](http://www.ncbi.nlm.nih.gov/entrez/query.fcgi?db=nucleotide&cmd=search&term=NM_001511) | 0.5 |
| FOXD1 | forkhead box D1 | [NM_004472](http://www.ncbi.nlm.nih.gov/entrez/query.fcgi?db=nucleotide&cmd=search&term=NM_004472) | 0.5 |
| GEM | GTP binding protein overexpressed in skeletal muscle | NM_181702 | 0.5 |
| IGF1R | insulin-like growth factor 1 receptor | [NM_000875](http://www.ncbi.nlm.nih.gov/entrez/query.fcgi?db=nucleotide&cmd=search&term=NM_000875) | 0.5 |
| KIF5C | kinesin family member 5C | [NM_004522](http://www.ncbi.nlm.nih.gov/entrez/query.fcgi?db=nucleotide&cmd=search&term=NM_004522) | 0.5 |
| NR4A2 | nuclear receptor subfamily 4, group A, member 2 | [NM_173173](http://www.ncbi.nlm.nih.gov/entrez/query.fcgi?db=nucleotide&cmd=search&term=NM_173173) | 0.5 |
| ODC1 | ornithine decarboxylase 1 | [NM_002539](http://www.ncbi.nlm.nih.gov/entrez/query.fcgi?db=nucleotide&cmd=search&term=NM_002539) | 0.5 |
| PALLD | rystal, cytoskeletal associated protein | [NM_016081](http://www.ncbi.nlm.nih.gov/entrez/query.fcgi?db=nucleotide&cmd=search&term=NM_016081) | 0.5 |
| PLAT | plasminogen activator, tissue | [NM_000931](http://www.ncbi.nlm.nih.gov/entrez/query.fcgi?db=nucleotide&cmd=search&term=NM_000931) | 0.5 |
| SEMA3C | sema domain, immunoglobulin domain (Ig), short basic domain, secreted, (rystalline) 3C | [NM_006379](http://www.ncbi.nlm.nih.gov/entrez/query.fcgi?db=nucleotide&cmd=search&term=NM_006379) | 0.5 |
| TNFRSF21 | tumor necrosis factor receptor superfamily, member 21 | [NM_014452](http://www.ncbi.nlm.nih.gov/entrez/query.fcgi?db=nucleotide&cmd=search&term=NM_014452) | 0.5 |
| WASF1 | WAS protein family, member 1 | [NM_003931](http://www.ncbi.nlm.nih.gov/entrez/query.fcgi?db=nucleotide&cmd=search&term=NM_003931) | 0.5 |
| DKK3 | dickkopf homolog 3 (Xenopus laevis) | [NM_015881](http://www.ncbi.nlm.nih.gov/entrez/query.fcgi?db=nucleotide&cmd=search&term=NM_015881) | 2 |
| MGLL | monoglyceride lipase | [NM_001003794](http://www.ncbi.nlm.nih.gov/entrez/query.fcgi?db=nucleotide&cmd=search&term=NM_001003794) | 2 |
| ANXA1 | annexin A1 | [NM_000700](http://www.ncbi.nlm.nih.gov/entrez/query.fcgi?db=nucleotide&cmd=search&term=NM_000700) | 2.1 |
| CAST | calpastatin | [NM_001042441](http://www.ncbi.nlm.nih.gov/entrez/query.fcgi?db=nucleotide&cmd=search&term=NM_001042441) | 2.1 |
| CD44 | CD44 molecule (Indian blood group) | [NM_000610](http://www.ncbi.nlm.nih.gov/entrez/query.fcgi?db=nucleotide&cmd=search&term=NM_000610) | 2.1 |
| CTLA4 | cytotoxic T-lymphocyte-associated protein 4 | [NM_005214](http://www.ncbi.nlm.nih.gov/entrez/query.fcgi?db=nucleotide&cmd=search&term=NM_005214) | 2.1 |
| MAP1B | microtubule-associated protein 1B | [NM_005909](http://www.ncbi.nlm.nih.gov/entrez/query.fcgi?db=nucleotide&cmd=search&term=NM_005909) | 2.1 |
| NCAM1 | neural cell adhesion molecule 1 | [NM_001076682](http://www.ncbi.nlm.nih.gov/entrez/query.fcgi?db=nucleotide&cmd=search&term=NM_001076682) | 2.1 |
| PDE4B | phosphodiesterase 4B, cAMP-specific (phosphodiesterase E4 dunce homolog, Drosophila) | [NM_002600](http://www.ncbi.nlm.nih.gov/entrez/query.fcgi?db=nucleotide&cmd=search&term=NM_002600) | 2.1 |
| ST8SIA1 | ST8 alpha-N-acetyl-neuraminide alpha-2,8-sialyltransferase 1 | [NM_003034](http://www.ncbi.nlm.nih.gov/entrez/query.fcgi?db=nucleotide&cmd=search&term=NM_003034) | 2.1 |
| EPHA4 | EPH receptor A4 | [NM_004438](http://www.ncbi.nlm.nih.gov/entrez/query.fcgi?db=nucleotide&cmd=search&term=NM_004438) | 2.2 |
| HMGCR | 3-hydroxy-3-methylglutaryl-Coenzyme A reductase | [NM_000859](http://www.ncbi.nlm.nih.gov/entrez/query.fcgi?db=nucleotide&cmd=search&term=NM_000859) | 2.2 |
| PAX6 | paired box gene 6 (aniridia, keratitis) | NM_001604 | 2.2 |
| ROCK2 | Rho-associated, coiled-coil containing protein kinase 2 | [NM_004850](http://www.ncbi.nlm.nih.gov/entrez/query.fcgi?db=nucleotide&cmd=search&term=NM_004850) | 2.2 |
| ADCYAP1 | adenylate cyclase activating polypeptide 1 (pituitary) | [NM_001117](http://www.ncbi.nlm.nih.gov/entrez/query.fcgi?db=nucleotide&cmd=search&term=NM_001117) | 2.3 |
| AMOT | angiomotin | [NM_133265](http://www.ncbi.nlm.nih.gov/entrez/query.fcgi?db=nucleotide&cmd=search&term=NM_133265) | 2.3 |
| CD74 | CD74 molecule, major histocompatibility complex, class II invariant chain | [NM_001025159](http://www.ncbi.nlm.nih.gov/entrez/query.fcgi?db=nucleotide&cmd=search&term=NM_001025159) | 2.3 |
| HBEGF | heparin-binding EGF-like growth factor | [NM_001945](http://www.ncbi.nlm.nih.gov/entrez/query.fcgi?db=nucleotide&cmd=search&term=NM_001945) | 2.3 |
| IGF2BP1 | insulin-like growth factor 2 mRNA binding protein 1 | [NM_006546](http://www.ncbi.nlm.nih.gov/entrez/query.fcgi?db=nucleotide&cmd=search&term=NM_006546) | 2.3 |
| LAMB3 | laminin, beta 3 | [NM_000228](http://www.ncbi.nlm.nih.gov/entrez/query.fcgi?db=nucleotide&cmd=search&term=NM_000228) | 2.3 |
| NTS | neurotensin | [NM_006183](http://www.ncbi.nlm.nih.gov/entrez/query.fcgi?db=nucleotide&cmd=search&term=NM_006183) | 2.3 |
| F2R | coagulation factor II (thrombin) receptor | [NM_001992](http://www.ncbi.nlm.nih.gov/entrez/query.fcgi?db=nucleotide&cmd=search&term=NM_001992) | 2.4 |
| HTATIP2 | HIV-1 Tat interactive protein 2, 30kDa | [NM_006410](http://www.ncbi.nlm.nih.gov/entrez/query.fcgi?db=nucleotide&cmd=search&term=NM_006410) | 2.4 |
| MTSS1 | metastasis suppressor 1 | [NM_014751](http://www.ncbi.nlm.nih.gov/entrez/query.fcgi?db=nucleotide&cmd=search&term=NM_014751) | 2.4 |
| NOV | nephroblastoma overexpressed gene | [NM_002514](http://www.ncbi.nlm.nih.gov/entrez/query.fcgi?db=nucleotide&cmd=search&term=NM_002514) | 2.4 |
| PREX1 | phosphatidylinositol 3,4,5-trisphosphate-dependent RAC exchanger 1 | [NM_020820](http://www.ncbi.nlm.nih.gov/entrez/query.fcgi?db=nucleotide&cmd=search&term=NM_020820) | 2.4 |
| SERPINF1 | serpin peptidase inhibitor, clade F (alpha-2 antiplasmin, pigment epithelium derived factor), member 1 | [NM_002615](http://www.ncbi.nlm.nih.gov/entrez/query.fcgi?db=nucleotide&cmd=search&term=NM_002615) | 2.4 |
| TRIB1 | tribbles homolog 1 (Drosophila) | [NM_025195](http://www.ncbi.nlm.nih.gov/entrez/query.fcgi?db=nucleotide&cmd=search&term=NM_025195) | 2.4 |
| FHL2 | four and a half LIM domains 2 | [NM_201557](http://www.ncbi.nlm.nih.gov/entrez/query.fcgi?db=nucleotide&cmd=search&term=NM_201557) | 2.5 |
| ROCK1 | Rho-associated, coiled-coil containing protein kinase 1 | [NM_005406](http://www.ncbi.nlm.nih.gov/entrez/query.fcgi?db=nucleotide&cmd=search&term=NM_005406) | 2.5 |
| SEMA3B | sema domain, immunoglobulin domain (Ig), short basic domain, secreted, (rystalline) 3B | [NM_001005914](http://www.ncbi.nlm.nih.gov/entrez/query.fcgi?db=nucleotide&cmd=search&term=NM_001005914) | 2.5 |
| SNAI2 | snail homolog 2 (Drosophila) | [NM_003068](http://www.ncbi.nlm.nih.gov/entrez/query.fcgi?db=nucleotide&cmd=search&term=NM_003068) | 2.5 |
| EPHB4 | EPH receptor B4 | [NM_004444](http://www.ncbi.nlm.nih.gov/entrez/query.fcgi?db=nucleotide&cmd=search&term=NM_004444) | 2.6 |
| VAV2 | vav 2 oncogene | [NM_003371](http://www.ncbi.nlm.nih.gov/entrez/query.fcgi?db=nucleotide&cmd=search&term=NM_003371) | 2.6 |
| NFATC2 | nuclear factor of activated T-cells, cytoplasmic, calcineurin-dependent 2 | [NM_012340](http://www.ncbi.nlm.nih.gov/entrez/query.fcgi?db=nucleotide&cmd=search&term=NM_012340) | 2.7 |
| ATP2B4 | ATPase, Ca++ transporting, plasma membrane 4 | [NM_001001396](http://www.ncbi.nlm.nih.gov/entrez/query.fcgi?db=nucleotide&cmd=search&term=NM_001001396) | 2.8 |
| S100B | S100 calcium binding protein B | [NM_006272](http://www.ncbi.nlm.nih.gov/entrez/query.fcgi?db=nucleotide&cmd=search&term=NM_006272) | 2.8 |
| L1CAM | L1 cell adhesion molecule | [NM_000425](http://www.ncbi.nlm.nih.gov/entrez/query.fcgi?db=nucleotide&cmd=search&term=NM_000425) | 2.9 |
| TFPI2 | tissue factor pathway inhibitor 2 | [NM_006528](http://www.ncbi.nlm.nih.gov/entrez/query.fcgi?db=nucleotide&cmd=search&term=NM_006528) | 2.9 |
| DPP4 | dipeptidyl-peptidase 4 (CD26, adenosine deaminase complexing protein 2) | [NM_001935](http://www.ncbi.nlm.nih.gov/entrez/query.fcgi?db=nucleotide&cmd=search&term=NM_001935) | 3 |
| LDLR | low density lipoprotein receptor (familial hypercholesterolemia) | [NM_000527](http://www.ncbi.nlm.nih.gov/entrez/query.fcgi?db=nucleotide&cmd=search&term=NM_000527) | 3 |
| TIAM1 | T-cell lymphoma invasion and metastasis 1 | [NM_003253](http://www.ncbi.nlm.nih.gov/entrez/query.fcgi?db=nucleotide&cmd=search&term=NM_003253) | 3.1 |
| TPR | translocated promoter region (to activated MET oncogene) | [NM_003292](http://www.ncbi.nlm.nih.gov/entrez/query.fcgi?db=nucleotide&cmd=search&term=NM_003292) | 3.1 |
| ITGB3 | integrin, beta 3 (platelet glycoprotein IIIa, antigen CD61) | [NM_000212](http://www.ncbi.nlm.nih.gov/entrez/query.fcgi?db=nucleotide&cmd=search&term=NM_000212) | 3.2 |
| LUM | lumican | [NM_002345](http://www.ncbi.nlm.nih.gov/entrez/query.fcgi?db=nucleotide&cmd=search&term=NM_002345) | 3.2 |
| EPHB1 | EPH receptor B1 | [NM_004441](http://www.ncbi.nlm.nih.gov/entrez/query.fcgi?db=nucleotide&cmd=search&term=NM_004441) | 3.6 |
| IGFBP2 | insulin-like growth factor binding protein 2 | [NM_000597](http://www.ncbi.nlm.nih.gov/entrez/query.fcgi?db=nucleotide&cmd=search&term=NM_000597) | 3.6 |
| KDR | kinase insert domain receptor (a type III receptor tyrosine kinase) | [NM_002253](http://www.ncbi.nlm.nih.gov/entrez/query.fcgi?db=nucleotide&cmd=search&term=NM_002253) | 3.7 |
| MMP8 | matrix metallopeptidase 8 (neutrophil collagenase) | [NM_002424](http://www.ncbi.nlm.nih.gov/entrez/query.fcgi?db=nucleotide&cmd=search&term=NM_002424) | 3.7 |
| KIT | v-kit Hardy-Zuckerman 4 feline sarcoma viral oncogene homolog | [NM_000222](http://www.ncbi.nlm.nih.gov/entrez/query.fcgi?db=nucleotide&cmd=search&term=NM_000222) | 3.9 |
| PRKAR1A | protein kinase, cAMP-dependent, regulatory, type I, alpha (tissue specific extinguisher 1) | [NM_212472](http://www.ncbi.nlm.nih.gov/entrez/query.fcgi?db=nucleotide&cmd=search&term=NM_212472) | 4.2 |
| MYO5B | myosin VB | NM_001080467 | 4.6 |
| NRCAM | neuronal cell adhesion molecule | [NM_001037132](http://www.ncbi.nlm.nih.gov/entrez/query.fcgi?db=nucleotide&cmd=search&term=NM_001037132) | 4.8 |
| CTSK | cathepsin K | [NM_000396](http://www.ncbi.nlm.nih.gov/entrez/query.fcgi?db=nucleotide&cmd=search&term=NM_000396) | 6.6 |
| RGS1 | regulator of G-protein rystalli 1 | [NM_002922](http://www.ncbi.nlm.nih.gov/entrez/query.fcgi?db=nucleotide&cmd=search&term=NM_002922) | 8.1 |
| CCL18 | chemokine (C-C motif) ligand 18 (pulmonary and activation-regulated) | [NM_002988](http://www.ncbi.nlm.nih.gov/entrez/query.fcgi?db=nucleotide&cmd=search&term=NM_002988) | 10 |
| CASP1 | caspase 1, apoptosis-related cysteine peptidase (interleukin 1, beta, convertase) | [NM_001223](http://www.ncbi.nlm.nih.gov/entrez/query.fcgi?db=nucleotide&cmd=search&term=NM_001223) | 14.2 |
| CYSLTR2 | cysteinyl leukotriene receptor 2 | [NM_020377](http://www.ncbi.nlm.nih.gov/entrez/query.fcgi?db=nucleotide&cmd=search&term=NM_020377) | 18.9 |
