# Supplementary material for: ADAR1-mediated regulation of melanoma invasion
Source: Nat Commun. 2018 May 31;9:2154. doi: 10.1038/s41467-018-04600-2 (PMC5981216; doi:10.1038/s41467-018-04600-2)
Supplement: Supplementary file 5 — Supplementary Data 2 [file 41467_2018_4600_MOESM5_ESM.docx]

**Supplementary Data 2: Primers**

| **primer** | **Sequence 5'🡪3'** |
| --- | --- |
| ITGB3 Fwd | 5' aaacccctgctatgatatgaagacc |
| ITGB3 Rev | 5' TTCAGAGACATCTTGCACTGTTTGAG |
| PAX6 Fwd | 5' CCACCACACCGGTTTCCTC |
| PAX6 Rev | 5' GGTGTTTGTGAGGGCTGTGTCT |
| GAPDH Fwd | 5'TGCACCACCAACTGCTTAGC |
| GAPDH Rev | 5' GGCATGGACTGTGGTCATGAG |
| ADAR1 Fwd | 5' acagccaaagacactccctctc |
| ADAR1 Rev | 5' ggctcagcatggctatctgg |
| P110-specific Fwd | 5' GGCAGCCTCCGGGTG |
| P110-specific Rev | 5' CTGTCTGTGCTCATAGCCTTGA 3' |
| P150-specific Fwd | 5' CGGGCAATGCCTCGC3' |
| P150-specific Rev | 5' AATGGATGGGTGTAGTATCCGC3' |
| HPRT F | 5'TGACACTGGCAAAACAATGCA |
| HPRT R | 5' GGTCCTTTTCACCAGCAAGCT |
| foxd1 f (Qrt) | 5' CTCGTATATCGCGCTCATCA |
| FOXD1 R (QRT) | 5' TCTTGACGAAGCAGTCGTTG |
| ADAR1-S DOM F | 5' GCTAAGCTTGCCGCCACCATGGCCGAGATCAAGGAGAAA |
| ΔCAT Rev | 5' GATCTCGAGCTAATGGTGATGGTGATGATGCACTGGGGTTACCTCTGTGAA |
| ITGB3 UTR I F | 5'GCTCTCGAGTAAGCAGTCATCCTCAGATCATTATCA |
| ITGB3 UTR-I R | 5'GTACGCGGCCCGCAGGGCCAGGTATGTGGAGGT |
| ITGB3 UTR II | 5'GCTCTCGAGTGCGGTTAAGATTCTCTGGG |
| ITGB3 UTR II R | 5' GTACGCGGCCGCCCAAGGCTTATTCTCAATGTTAACA |
| miR-211 F | 5' GTA CGC GGC CGC CAG AGT GGG TAG AAT GGG GAT |
| miR-211 R | 5' GTG AAT TCC AAC CCA AGC AAG GAT TGA TAT T |
| miR-22 F | 5' GTA CGC GGC CGC CCT GCA TTA GAA TCT CAG AGG TCT AC |
| miR-22 R | 5' GTG AAT TCC CAA AAC GTA TCA TCC ACC CT |
| miR-185 F | 5’gcatgcggccgcaaaggcaaggtcacaggtc |
| miR-185 R | 5’ggaattctaaacagatctccggacagc |
| miR-138-1 F | 5’ GTACGCGGCCGCAGTTCACAAAAGGCACAACGA |
| miR-138-1 R | 5’ GTGAATTCCAGGAGGCCAGTGTCTGC |
| miR-138-2 F | 5’GTACGCGGCCGCTGAGCCCTGGTGCCATACT |
| miR-138-2 R | 5’GTGAATTCGGTGATTCTGAGGAGCAGTCTG |
| miR-22 UTR SDM-A F | 5'CTC AGG CCT GAC TCT CAG TAA CGA TGG TAG GAA CTG CTG GG |
| miR-22 UTR SDM-A R | 5'CCC AGC AGT TCC TAC CAT CGT TAC TGA GAG TCA GGC CTG AG |
| miR-22 UTR SDM-B F | 5'ACC CTT CAG ATT TGC CTT ATT GTC CGA TCT ACT CTG GAG GTT TGT TTA G |
| miR-22 UTR SDM-B R | 5'CTA AAC AAA CCT CCA GAG TAG ATC GGA CAA TAA GGC AAA TCT GAA GGG T |
| miR-211 UTR SDM-A F | 5' CCC AGC TAT GGT TCT CTC GCA CGT GGA GTC CTT GCA AGC TAA |
| miR-211 UTR SDM-A R | 5' TTA GCT TGC AAG GAC TCC ACG TGC GAG AGA ACC ATA GCT GGG |
| miR-211 UTR SDM-B F | 5' GAT GTC TCT CAT GAC CAA ATG CTT TTC CTC ACA TGT AGA GAG TGC TAT TGT A |
| miR-211 UTR SDM-B R | 5' TAC AAT AGC ACT CTC TAC ATG TGA GGA AAA GCA TTT GGT CAT GAG AGA CAT C |
| miR-138 UTR SDM F | 5'TCA TTC ATG GCC TGG GGG ATG TAT CCG TAT CTC CCA GTT CAT AAT C |
| miR-138 UTR SDM R | 5'GAT TAT GAA CTG GGA GAT ACG GAT ACA TCC CCC AGG CCA TGA ATG A |
| miR-185 SDM F | 5'CTT TCT CAA TGA AAG TCT CAT TCT ATC CTA TAT ACA AAC CCG TTT TCC AAC ATT TGT TAA TAG |
| miR-185 R | 5'CTA TTA ACA AAT GTT GGA AAA CGG GTT TGT ATA TAG GAT AGA ATG AGA CTT TCA TTG AGA AAG |
| miR-22 Pr. F | 5'gctCTCGAGGAAGGAAAAGGCATGGATTTC |
| miR-22 Pr. R | 5'gctAAGCTTGGAGCAAATCACTGCGTCC |
| miR-22 Pr. MUT F | 5'-gctctgtgagttggcagttgtcaactgagctcggaatccagcc |
| miR-22 Pr. MUT R | 5'-ggctggattccgagctcagttgacaactgccaactcacagagc |
| ITGB3 Pr. F | 5'TACGAGCTCGGTGGAGGATTGTCAGAAAAAA |
| ITGB3 Pr. R | 5'TACGCTAGCGCTCGCATCTCGTCCGCCT |
| ITGB3 Pr. MUT F | 5'cattcacccactacctcgagtttcattcgttaacattgtcattcatttgactggcaaacatctgctga |
| ITGB3 Pr. MUT R | 5'tcagcagatgtttgccagtcaaatgaatgacaatgttaacgaatgaaactcgaggtagtgggtgaat |
| Pri-miR-22 F | 5' CCTTTCCCTTAGGAGCCTG |
| Pri-miR-22 R | 5'CAGAGGGCAACAGTTCTTCA |
| FOXD1 F (clon) | 5'ATCACCGGTGCCACCATGACCCTGAGCACTGAGATG |
| FOXD1 R (clon) | 5'GCGAATTCTTAACAATTGGAAATCCTAGCAGTAA |
| miR-22 EDT F | 5' AGCCTGTTCCTCTCACGCC |
| miR-22 EDT R | 5' TTGGGTTGTTTGAGCCTTCTACTC |
| miR-22 EDT SEQ | 5' CAATCCAGCCAGTGTCTCTAATCT |
| ADAR1 K554E Fwd | 5' GGAAGCGAGAAAGTGGCCAA |
| ADAR1 K554E Rev | 5' TTGGCCACTTTCTCGCTTCC |
| ADAR1 K665E Fwd | 5' TCCCAGCGAGAAAGTGGCAA |
| ADAR1 K665E Rev | 5' TTGCCACTTTCTCGCTGGGA |
| ADAR1 K776E Fwd | 5' CACAGCGAGAAGCAAGGCAA |
| ADAR1 K776E Rev | 5' TTGCCTTGCTTCTCGCTGTG |
| Stau1 Fwd | 5’actcactatagggagacccaagcttATGTCTCAAGTTCAAGTGCAAG |
| Stau1 Rev | 5’agggccctctagatgcatgctcgagTCAGCACCTCCCACACAC |
| ChIP ITB3 Promoter Fwd | CTTGCCCTCAACAGGTAGGTAGT |
| ChIP IGB3 Promoter Rev | CGCTGGATTCTTGGGACACAT |
| ChIP ITGB3 coding Fwd | AAGTCCTTTACCATAAAGCCCG |
| ChIP ITGB3 coding Rev | GAAGGGCGATAGTCCTCCTC |
